# Supplementary figures and images for: The ribosomal RNA m5C methyltransferase NSUN-1 modulates healthspan and oogenesis in Caenorhabditis elegans
Source: eLife. 2020 Dec 8;9:e56205. doi: 10.7554/eLife.56205 (PMC7746234; doi:10.7554/eLife.56205)

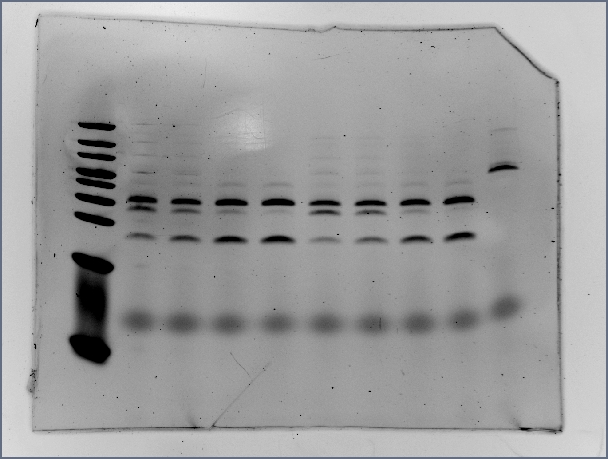

Supplement: Figure 1—source data 1. — ZIP-compressed archive containing two gel images, which were used for quantification, and a xlsx-file with the calculated methylation values. [file elife-56205-fig1-data1.zip › COBRA_replicate_1_2.jpg]

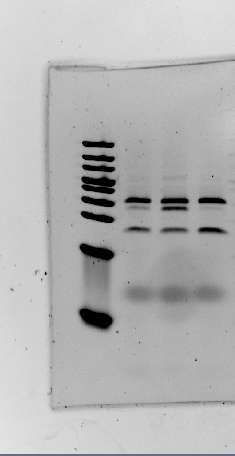

Supplement: Figure 1—source data 1. — ZIP-compressed archive containing two gel images, which were used for quantification, and a xlsx-file with the calculated methylation values. [file elife-56205-fig1-data1.zip › COBRA_replicate_3.jpg]
